# Single-Epoch, Single-Frequency Multi-GNSS L5 RTK under High-Elevation Masking

**DOI:** 10.3390/s19051066

**Published:** 2019-03-02

**Authors:** Kan Wang, Pei Chen, Peter J. G. Teunissen

**Affiliations:** 1 Department of Spatial Sciences, Curtin University, GPO Box U1987, Perth, WA 6845, Australia; kan.wang@curtin.edu.au (K.W.); P.Teunissen@curtin.edu.au (P.J.G.T.); 2 School of Astronautics, Beihang University, Beijing 100191, China; 3 Department of Geoscience and Remote Sensing, Delft University of Technology, Building 23, Stevinweg 1, 2628 CN Delft, The Netherlands

**Keywords:** L5 frequency, multi-GNSS, single-epoch ambiguity resolution, instantaneous positioning, high elevation masking

## Abstract

The Japanese Quasi-Zenith Satellite System (QZSS) satellite system has placed in orbit four satellites by October 2017. The Indian Regional Navigation Satellite System (IRNSS) system has launched the new satellite IRNNSS-11 in April 2018, completing seven operational satellites. Together with the GPS block IIF satellites and the Galileo satellites, four different global navigation satellite systems (GNSSs) are providing precise L5 signals on the frequency of 1176.45 MHz. In this contribution, we challenge the strength of the multi-GNSS model by analysing its single-frequency (L5), single-epoch (instantaneous) precise positioning capabilities under high-elevation masking (up to 40 degrees). With more satellites available, multi-GNSS real time kinematic (RTK) positioning is possible using L5-only signals with a high customary elevation mask. This helps to enable positioning in areas with constrained measurement geometry, and could significantly reduce the multipath effects in difficult measurement environments like urban canyons and mountainous areas. In this study, benefiting from the location of the Asia–Australia area, instantaneous multi-GNSS L5 RTK analysis is performed with respect to the ambiguity resolution and positioning performance. Formal results are shown and discussed for baselines located in different grids covering Australia, part of the Pacific Ocean, Indian Ocean and Asia, and empirical analysis is given for two baselines in Perth, Australia. Compared to the stand-alone cases, for baselines in Perth, it is shown that combining L5 signals from GPS/Galileo/QZSS/IRNSS significantly improves both the ambiguity success rates (ASR) and the positioning performance under high elevation mask. While the average single-system ASR is under 50% even with a low elevation mask of 10 degrees, combining all the four systems increases the ASR to above 95% under an elevation cut-off angles of 40 degrees. With an elevation mask of 40 degrees, using satellites from one system does not allow for meaningful positioning solutions of more than 8 h within the test day, while mm-to-cm level ambiguity-fixed standard deviations could be obtained based on the positioning results of almost the entire day when combining all the four systems. In addition to that, simulation was also performed for receivers with larger signal standard deviations, i.e., for low-cost receivers or receivers located in environments with larger multipath.

## 1. Introduction

In April 2018, the Indian Regional Navigation Satellite System (IRNSS) launched the satellite IRNSS-1I to replace its first satellite IRNSS-1A with failed on-board atomic clocks [1,2]. This inclined geosynchronous orbit (IGSO) satellite was added to the IRNSS constellation, completing seven operational satellites providing signals on L5 (1176.45 MHz), including three satellites on IGSO orbits and three geostationary orbit (GEO) satellites. In the meantime, the Japanese Quasi-Zenith Satellite System (QZSS) has also increased its number of satellites to four by October 2017 [3]. With three quasi-zenith orbit (QZO) satellites and one GEO satellite flying over Japan with high elevation angles, triple-frequency signals on L1, L2 and L5 are provided to users in Asia-Oceania region as a GPS augmentation [4]. By 13 August 2018, apart from QZSS and IRNSS, 12 GPS block IIF satellites and 19 Galileo In-Orbit Validation (IOV) and full operational capability (FOC) satellites are also transmitting operational signals on L5 and E5a of 1176.45 MHz, respectively [5,6]. Among them, the Galileo Satellites E14 and E18 were launched to incorrect orbits but recovered [7,8].

**Figure 1:**
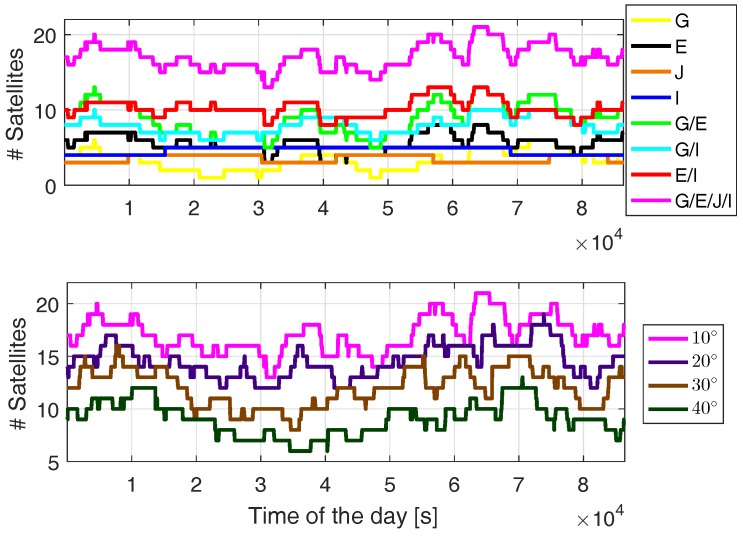
Number of visible satellites from different systems with an elevation mask of 10 degrees (**top**) and from all the four systems with different elevation masks (**bottom**). The figures were generated based on the MGEX combined broadcast ephemeris on DOY 224, 2018 for station CUAA located in Perth, Australia. G, E, J and I are denoted as system identifications for GPS, Galileo, QZSS and IRNSS, respectively.

Benefiting from the location of Australia, the L5 signals can be received from all the four systems. As an example, Figure 1 shows the number of visible satellites for station CUAA located in Curtin University, Perth, Australia on day of year (DOY) 224, 2018. The number of visible satellites from different systems above an elevation mask of 10 degrees and the total number of visible satellites above different elevation masks are shown in the top and bottom panels, respectively. In this contribution, G, E, J and I are denoted as system identifications for GPS, Galileo, QZSS and IRNSS, respectively. The figure was generated based on the combined Multi-GNSS Experiment (MGEX) broadcast ephemeris [9,10,11] on the same day. Note that the IRNSS-1I launched in April 2018 and the QZSS satellite QZS-3 (J07) were not contained in the MGEX broadcast ephemeris on this day. Since J07 is a GEO satellite, to simulate its approximate positions in WGS84, we used its positions on DOY 154, 2018, i.e., the nearest day before DOY 224, 2018 with J07 available in the MGEX broadcast ephemeris. It can be observed that, even under a high elevation mask of 40 degrees, the total number of satellites remains equal or above 8 more than 80% of the time.

**Figure 2:**
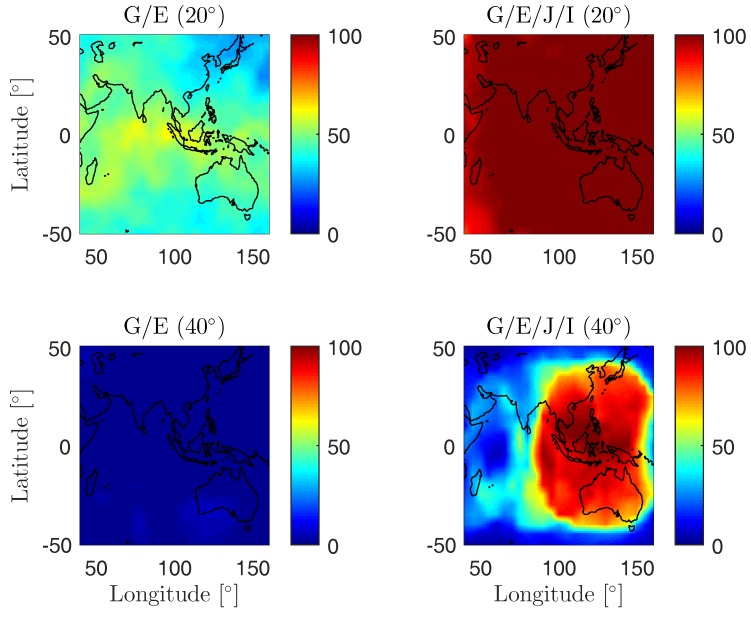
Percentages with the 24 h period that at least eight satellites are above the elevation mask of 20 (**top**) and 40 degrees (**bottom**). The figure was generated based on the MGEX combined broadcast ephemeris on DOY 224, 2018 with a data sampling interval of 30 s. The newly launched I09 and the QZSS satellite J07 were not contained in the broadcast ephemeris. The satellite positions (WGS84) of J07 on DOY 154, 2018 were used for the computation.

The benefits of combining different GNSSs for L5 signal processing are further illustrated in Figure 2, which shows the percentages within a 24 h period that at least eight satellites are above the elevation mask of 20 (top) and 40 degrees (bottom) on DOY 224, 2018. Satellite positions (WGS84) of J07 on DOY 154, 2018 were used for the plots. We see that increasing the elevation mask reduces the percentage values, and, for the major part of the area, combining satellites from more systems is essential for the increase in percentages.

In recent years, the QZSS and/or IRNSS data were used in stand-alone cases or combined with other systems to achieve precise real time kinematic (RTK) positioning results. Stand-alone RTK performance studies were performed in [12,13] for IRNSS (six satellites) and triple-frequency QZSS (four satellites). Combined with each other or with other GNSSs like GPS, Galileo and Beidou Navigation Satellite System (BDS), the ambiguity resolution and positioning performance were also studied in [14,15,16,17,18].

In poor measurement environments like mountainous areas and urban canyons, signals from low-elevation satellites may not be received due to constrained geometry or can only be received with the presence of large multipath effects. As pointed out by [16,19], combining satellite systems is then essential to improve the ambiguity resolution and positioning performance for high-elevation masked instantaneous RTK. The instantaneous, single-frequency multi-GNSS RTK performances with the four-system common frequency of IRNSS, QZSS, GPS and Galileo for different elevation masks has however not yet been investigated. Next to the robustness against elevation-masking, our emphasis is hereby also on *instantaneous* as this is under the given circumstances the most challenging case, with the important additional advantage that the positioning results will then be immune for carrier-phase cycle slips.

In this contribution, we first perform formal analysis for a large area covering Australia, part of the Indian Ocean, Pacific Ocean and Asia. The ambiguity resolution and positioning performances are discussed for single-epoch L5 RTK using different system combinations and elevation masks. After that, using two baselines in Perth, Australia, we show and compare the empirical and formal results based on real data tracking. In addition to that, discussion is also performed for larger signal standard deviations of low-cost receivers or receivers in environments with larger multipath. We remark that, in this study, we put our focus on L5-only RTK performance. This is not only because of the good L5 code signal precision, but also because the IRNSS satellites, which are of importance for users in Australia, Asia-Pacific area and Indian Ocean, only send signals on L5 and S-band [20].

In the next section, we give an overview of our processing strategy. After introducing the measurement set up, signal analysis is performed for multi-GNSS L5 signals. After that, ambiguity resolution and the positioning performance are assessed based on formal and empirical analysis. Results under different elevation masks and using different system combinations are compared and discussed. Subsequently, formal analysis is performed for larger signal standard deviations. A summary with conclusions is provided in the last section.

## 2. Processing Strategy

For relative positioning using short baselines within several kilometres, the atmospheric delays are assumed to be largely reduced by formulating differences between-receivers. The unknown variables that remain to be estimated are baseline increments and the double-difference (DD) ambiguities. The short baseline single-frequency DD model with phase (Δϕ) and code (Δp) observed-minus-computed (O-C) terms can be formulated as:

(1) $$E\left[\begin{array}{c} \Delta \phi \\ \Delta p \end{array}\right]=\left[\begin{array}{cc} D_{m}^{T}A & \lambda_{j}I_{m-1}\\ D_{m}^{T}A & 0 \end{array}\right]\left[\begin{array}{c} \Delta b\\ a \end{array}\right],$$

where *m* denotes the number of tracked satellites. The m×3 matrix *A* is equal to ${\left[u^{1},\cdots ,u^{m}\right]}^{T}$, for which $u^{s}$ represents the three-dimensional satellite-to-receiver unit vector from satellite *s* to the rover. The differencing operator $D_{m}^{T}=\left[-e_{m-1},I_{m-1}\right]$ forms the double-differences of *A*, where $e_{m-1}$ and $I_{m-1}$ represent vector of ones and identify matrix with the size of m−1, respectively. The DD ambiguity vector *a* is given in cycles with the wavelength on frequency *j* denoted by $\lambda_{j}$. The baseline increment vector is denoted as Δb, and E[·] is the expectation operator. In this study, the combined MGEX broadcast ephemeris [9,10,11] is used to compute the satellite positions. For each epoch, the satellite with the highest elevation angle is selected as the reference satellite for all systems, which is denoted by the superindex 1. Since the receiver and antenna types are the same for all stations in this study, the differential inter-system biases (ISBs) are assumed to be zero [21]. Note that tables of variables and acronyms are given in the Appendix A.

Assuming that the variance matrices of the undifferenced phase and code observations are elevation-dependent diagonal matrices, the dispersion of Equation (1) can be formulated as:

(2) $$D\left[\begin{array}{c} \Delta \phi \\ \Delta p \end{array}\right]=\left[\begin{array}{cc} 2D_{m}^{T}Q_{\phi }D_{m} & 0\\ 0 & 2D_{m}^{T}Q_{p}D_{m} \end{array}\right],$$

where D[·] denotes the dispersion operator, and $Q_{\phi }$ and $Q_{p}$ are given as

(3) $$\begin{array}{ccc} Q_{\phi } & = & \mathrm{diag}(\sigma_{\phi_{k(1)}}^{2}{\left(w^{1}\right)}^{-1},\cdots ,\sigma_{\phi_{k(m)}}^{2}{\left(w^{m}\right)}^{-1}), \end{array}$$

(4) $$\begin{array}{ccc} Q_{p} & = & \mathrm{diag}(\sigma_{p_{k(1)}}^{2}{\left(w^{1}\right)}^{-1},\cdots ,\sigma_{p_{k(m)}}^{2}{\left(w^{m}\right)}^{-1}), \end{array}$$

where the diagonal elements of the diagonal matrix diag(·) are contained in (·). The terms $\sigma_{\phi_{k(s)}}$ and $\sigma_{p_{k(s)}}$ are zenith-referenced undifferenced standard deviations of the phase and code observations, respectively, for system k(s). The term k(s) denotes the system number of satellite *s*. The elevation-dependent exponential weighting function $w^{s}$ for satellite *s* is given as [22]:

(5) $$w^{s}={\left(1+10\cdot \exp\left(-\frac{{\mathrm{ele}}^{s}}{10}\right)\right)}^{-2},$$

where ${\mathrm{ele}}^{s}$ denotes the elevation angle from the receiver to satellite *s* in degrees.

In this study, single-epoch processing is performed based on least-squares adjustment using L5 code and phase signals with different system combinations [18] and different elevation masks. We remark again that, in single-epoch processing, the ambiguities are estimated independently for each epoch, thus making the results immune to carrier-phase cycle slips. The DD ambiguities and baseline increments are estimated for each epoch independently without any temporal constraints, thus realizing a truely kinematic setup. In the next section, the measurement setup will be introduced before we perform the signal analysis.

## 3. Measurement Setup and Signal Analysis

**Figure 3:**
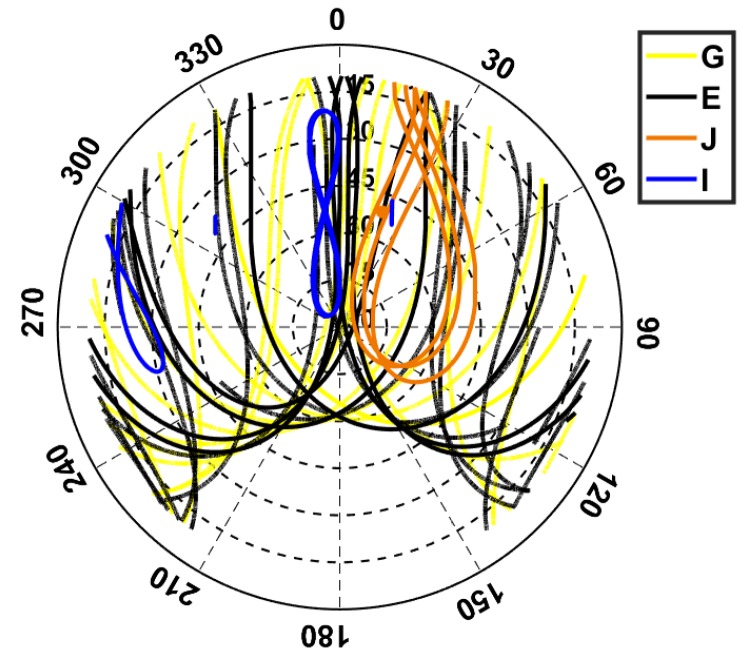
Skyplot for station CUAA on DOY 224, 2018. An elevation mask of 10 degrees was set for the plot.

In this contribution, for the signal analysis and real data analysis, 1 Hz data on L5 (1176.45 MHz) was collected from two baselines located in Curtin University, Perth, Australia, i.e., the baselines CUAA-CUCC and CUAA-CUBB. The three stations have the same receiver type JAVAD TRE_G3TH DELTA (Javad, San Jose, CA, USA) and the same antenna type TRM59800.00 SCIS (Trimble, Sunnyvale, CA, USA). As an example, Figure 3 shows the skyplot for station CUAA on DOY 224, 2018 with an elevation mask of 10 degrees. Note that the skyplot was generated purely based on the MGEX broadcast ephemeris and the ground truth of the station coordinates, and the satellite positions (WGS84) of J07 on DOY 154, 2018 were used for the plot.

**Figure 4:**
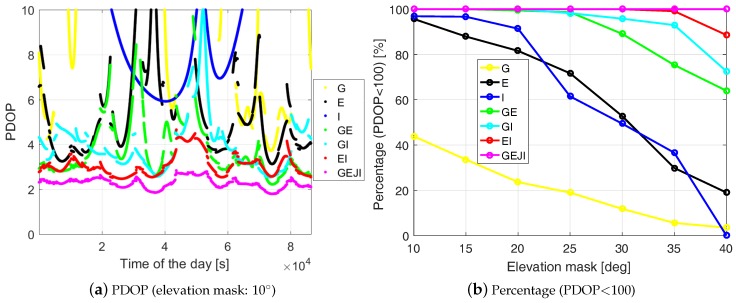
PDOP (Equation (6)) with an elevation mask of 10 degrees (**a**) and the percentages within a 24 h period that PDOP is smaller than 100 (**b**). The ground truth of baseline CUAA-CUCC and the satellite geometry on DOY 224, 2018 were used for the plots. Satellite positions (WGS84) of J07 on DOY 154, 2018 were used for the plots. The data sampling rate is 1 Hz. Note that (**a**) is zoomed out to 0–10.

To illustrate the changes of the measurement geometry under different elevation masks and using different system combinations, Figure 4a shows the Position Dilution of Precision (PDOP) of baseline CUAA-CUCC on DOY 224, 2018 under an elevation mask of 10 degrees, and Figure 4b shows the percentages within the 24 h period that PDOP is smaller than 100 for different elevation masks and system combinations. The PDOP is a measure to evaluate the influences of the measurement geometry on the positioning precision. For a DD scenario, the PDOP is given as [13]

(6) $$\mathrm{PDOP}=\sqrt{\mathrm{tr}\{{\left(A^{T}D_{m}{\left(D_{m}^{T}W^{-1}D_{m}\right)}^{-1}D_{m}^{T}A\right)}^{-1}\}},$$

with tr{·} denoting the trace of the matrix in {·}, and the weight matrix *W* is defined as

(7) $$W=\mathrm{diag}(w^{1},\cdots ,w^{m}).$$

We remark that Figure 4 is generated purely based on the geometry, and the time epochs with PDOPs larger than 100 were not used for the plots in Figure 4. The satellite positions (WGS84) of J07 on DOY 154, 2018 were used for the computation. We see that the PDOPs in combined cases are in general lower than those in stand-alone cases. From Figure 4b, it can be observed that, under a high elevation mask of 30 degrees or beyond, combining different GNSSs is essential to maintain the PDOPs below 100 in most of the time. Even with an elevation mask of 40 degrees, combining all the four systems allows the PDOP to stay below 100 in the entire day, and below 10 in more than 90% of the time on the test day.

The L5 phase ($\sigma_{\phi }$) and code standard deviations ($\sigma_{p}$) are computed with the least-squares variance component estimation (LS-VCE) procedure [23] using the DD residuals, which were obtained by subtracting the DD ambiguity part (for phase) and the DD geometric distance with known baseline coordinates from the DD observations. The known ambiguities were obtained with the strong baseline-known model, i.e., introducing known baseline coordinates with only ambiguities to be fixed [13,18]. In Table 1, the phase and code standard deviations are given for both baselines with the elevation mask set to be 10 and 40 degrees. Note that the multipath effects were not corrected for the calculated standard deviations. Data on DOY 223, 2018 was used for calculating the standard deviations of GPS/Galileo/IRNSS, and data on DOY 153, 2018 was used for calculation of the QZSS standard deviations, when all the four QZSS satellites were contained in the MGEX broadcast ephemeris. It can be observed that the signal standard deviations under a high elevation mask of 40 degrees are in general smaller than those under an elevation mask of 10 degrees. This corresponds to the fact that the change of the multipath does not totally correspond to the elevation-weighting function given in Equation (5). The multipath effects mainly influence signals with low elevations.

## 4. Formal Analysis

In this section, formal analysis is performed to assess the ambiguity resolution and positioning performance of short baselines in a large area covering Australia, part of Asia, Indian Ocean and Pacific Ocean under different elevation masks. Satellites from different GNSSs sending L5 signals are used for the analysis. Note that the formal analysis in this section is purely based on the baseline and the satellite geometry on DOY 224, 2018, while the WGS84 positions of the GEO satellite J07 are taken from DOY 154, 2018.

### 4.1. Ambiguity Resolution

**Figure 5:**
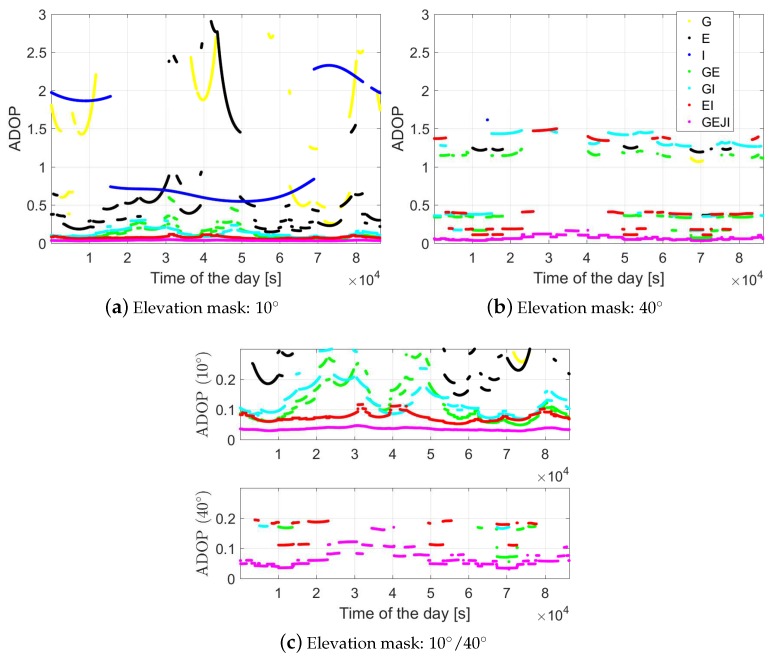
ADOP (Equation (10)) of baseline CUAA-CUCC on DOY 224, 2018 with elevation masks of 10 (**a**); 40 degrees (**b**) and the figures zoomed out for small ADOP values (**c**). The data sampling rate is 1 Hz.

With the least-squares adjustment theory, the float ambiguities are estimated together with the baseline increments using the DD O-C terms (Equation (1)) and the corresponding variance matrices (Equation (2)). In this contribution, our ambiguity resolution is based on applying the easy-to-compute integer bootstrapping (IB) estimator to the LAMBDA-decorrelated float ambiguities [24]. The IB ambiguity success-rate (ASR) ($P_{\mathrm{IB}}$), which lower bounds the integer least-squares (ILS) ASR ($P_{\mathrm{ILS}}$), can be calculated as [25,26]:

(8) $$P_{\mathrm{ILS}}\geq P_{\mathrm{IB}}=\prod_{i=1}^{m-1}\left(2\Phi \left(\frac{1}{2\sigma_{{\hat{z}}_{i|K}}}\right)-1\right)$$

with Φ(x) as the cumulative distribution function (CDF) of the standard normal distribution:

(9) $$\Phi \left(x\right)=\int_{-\infty }^{x}\frac{1}{\sqrt{2\pi }}\exp\left(-\frac{y^{2}}{2}\right)dy,$$

where the *i*-th conditional standard deviation of the decorrelated ambiguity is denoted by $\sigma_{{\hat{z}}_{i|K}}$ with K=1,⋯,i−1. As an easy-to-compute scalar, the ambiguity dilution of precision (ADOP) measures the model strength of successful ambiguity resolution [27], which is given in cycles as:

(10) $$\mathrm{ADOP}={\sqrt{|Q_{\hat{a}\hat{a}}|}}^{\frac{1}{m-1}},$$

where $Q_{\hat{a}\hat{a}}$ denotes the variance-covariance matrix of the float ambiguities. To have an overview of the ambiguity resolution performance, in Figure 5, the ADOP of baseline CUAA-CUCC with elevation masks of 10 and 40 degrees are illustrated for different system combinations. Only time epochs with not less than four satellites and with PDOP smaller than 100 were considered as valid and were used for the plots. The signal standard deviations with the corresponding elevation masks given in Table 1 were used for the computation. We see that, under an elevation mask of 40 degrees, there are almost no valid time points for stand-alone cases due to the poor measurement geometry. In contrast to that, under the same elevation mask of 40 degrees, combining all the four systems allows the ADOP to remain below 0.3 cycles in the entire day and below 0.12 cycles above 90% of the time.

**Figure 6:**
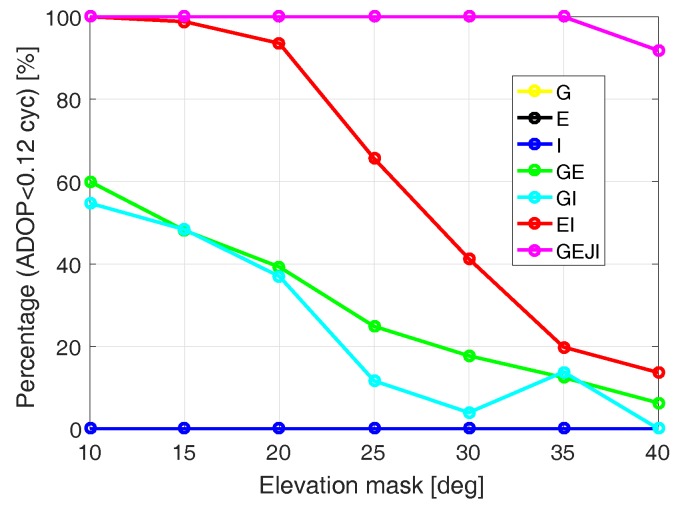
Percentages within a 24 h period that ADOP is smaller than 0.12 cycles for baseline CUAA-CUCC on DOY 224, 2018. Note that the yellow and the black lines are overwritten by the blue line. The data sampling rate is 1 Hz. The yellow and black lines are overwritten by the blue line.

According to [28], an ADOP of 0.12 cycles approximately corresponds to a formal ILS ASR of 99.9%. For the same baseline CUAA-CUCC, Figure 6 gives an overview of the percentages within the 24 h period of DOY 224, 2018 that ADOP is smaller than 0.12 cycles for different system combinations and elevation masks. Note that the ADOPs are calculated using signal standard deviations under the corresponding elevation masks, and the yellow and black lines are overwritten by the blue line. It can be observed that combining different systems is important for maintaining the ADOP at low values, and the number of the required systems increases with the elevation mask. Combining all the four systems, the ADOP is below 0.12 cycles during the entire day even with a high elevation mask of 35 degrees. The increasing percentage value in GPS/IRNSS-combined case from the elevation mask of 30 to 35 degrees is caused by fact that the signal standard deviations have decreased from 30 to 35 degrees, while the influence of the geometry change between these two elevation masks is not significant enough for time points with ADOP below 0.12 cycles.

**Figure 7:**
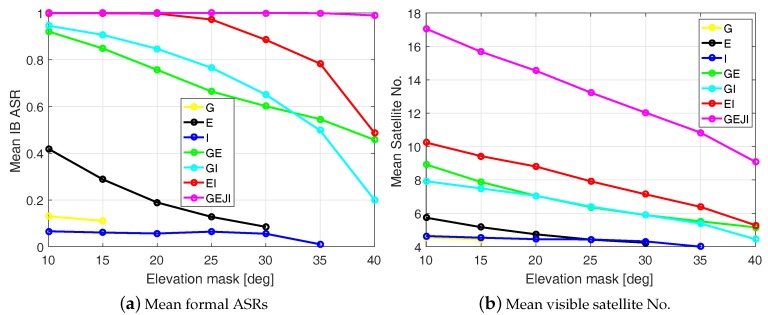
Mean formal IB ASRs (**a**) and the corresponding mean visible satellite numbers (**b**) for baseline CUAA-CUCC on DOY 224, 2018. The data sampling rate is 1 Hz.

Based on Equation (8), the mean formal IB ASR is computed for baseline CUAA-CUCC on DOY 224, 2018 using different system combinations and under different elevation masks (see Figure 7a). Note that only time epochs with at least four satellites and with PDOP smaller than 100 are considered as valid time points, and the mean formal ASR is the average value of the formal ASRs of all valid time points. If the length of the valid time points is shorter than 8 h within the day, the result is considered to be not representative and not shown in the plot. Again, we see that no representative mean ASRs can be produced in stand-alone cases under the elevation mask of 40 degrees, while combining all the four systems allows a mean IB ASR of almost 100% under such a high elevation mask. For the representative mean formal ASRs, the mean visible satellite numbers are also correspondingly shown in Figure 7b using the valid time points. The general trend of the decreasing mean ASRs with the increasing elevation masks and decreasing number of systems can also be observed in the mean visible satellite numbers.

**Figure 8:**
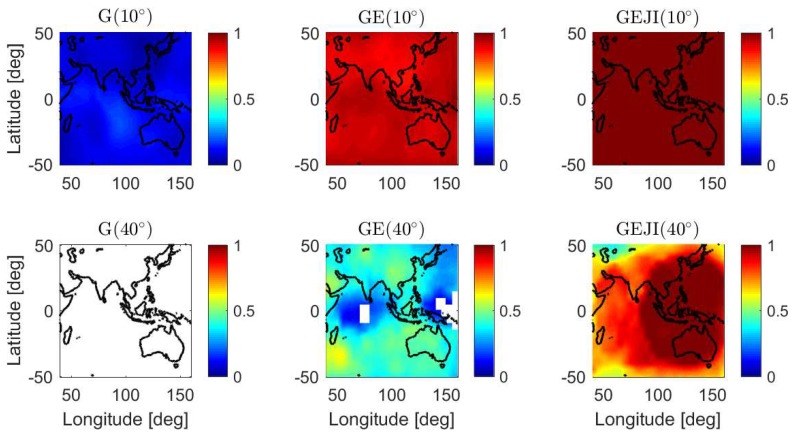
Mean formal IB ASRs for short baselines from 40${}^{\circ }$ E to 160${}^{\circ }$ E and from 50${}^{\circ }$ S to 50${}^{\circ }$ N on DOY 224, 2018. The data sampling interval is 30 s.

In addition to the baseline in Perth, an overview of the L5 mean ASRs is also given for a larger area including Australia, part of the Pacific Ocean, the Indian Ocean and Asia. The mean formal IB ASRs of short baselines located in grids from 40${}^{\circ }$ E to 160${}^{\circ }$ E and from 50${}^{\circ }$ S to 50 ${}^{\circ }$ N are computed with data sampling interval of 30 s and shown in Figure 8. The satellite geometry on DOY 224, 2018 was used for the computation, and the same criterion as before was used to compute representative results. The signal standard deviations of baseline CUAA-CUCC (Table 1) were used for the calculation. If no representative value can be generated for a baseline, i.e., with valid time points shorter than 8 h within the day, the corresponding grid is illustrated in white. From Figure 8, it can be observed that, under the elevation mask of 40 degrees (bottom panels), the GPS stand-alone case cannot obtain valid L5 ASRs longer than 8 h within the test day, and combining four systems effectively improves the mean ASRs to almost 100% in most of Australia and the southeast Asia (see the right bottom panel of Figure 8).

### 4.2. Positioning Performance

**Figure 9:**
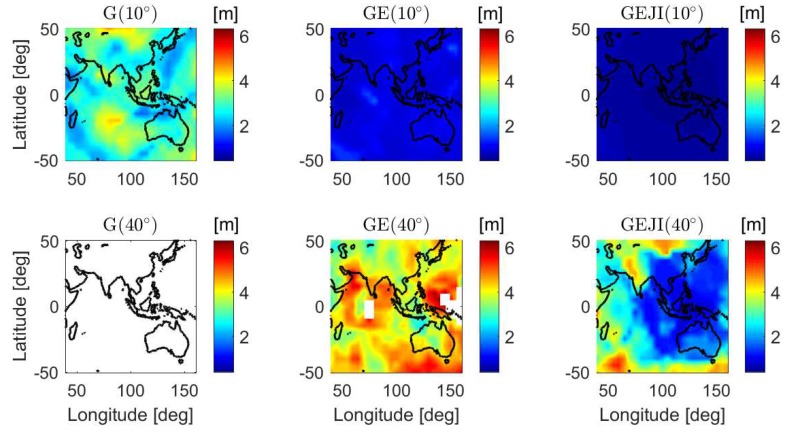
Average formal standard deviations of the ambiguity-float height errors on DOY 224, 2018. The data sampling interval is 30 s.

Together with the mean ASRs, the average formal standard deviations of the baseline errors are also computed for the same area as in Figure 8 using the same satellite geometry. The average formal standard deviations of the baseline errors are computed as the square roots of the mean formal variances of the baseline errors. As before, only valid time points with at least four satellites and PDOP smaller than 100 contribute to the standard deviations, and a lower time limit of 8 h is set for representative results. The sampling interval of the data is 30 s. In Figure 9, the average formal standard deviations of the ambiguity-float height errors are shown as examples. We see that combing different systems improves the ambiguity-float solutions under both the low and high elevation masks, while increasing the number of the combined systems has significant benefits under the high elevation mask of 40 degrees. In GPS stand-alone case, as shown in the left bottom panel of Figure 9, valid L5 single-epoch solutions cannot be computed in more than 8 h of the test day under the elevation mask of 40 degrees, while combining all the four systems (see the right bottom panel of Figure 9) delivers average height standard deviations below 2 m in above 45% of test area.

**Figure 10:**
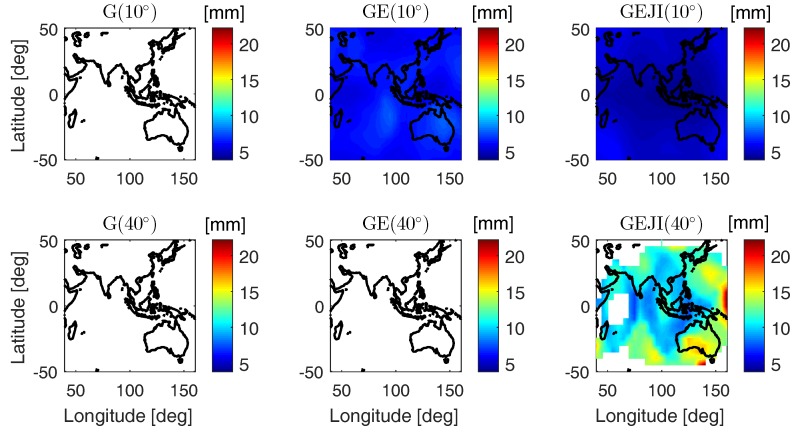
Average formal standard deviations of the ambiguity-fixed height errors on DOY 224, 2018. The data sampling interval is 30 s.

The average height formal standard deviations are also computed for the ambiguity-fixed case in Figure 10. Only the valid time points with ASR larger than 99.9% contribute to the ambiguity-fixed solutions, and the lower time limit of 8 h is set as before. As shown in Figure 10, under an elevation mask of 40 degrees, GPS stand-alone case and GPS/Galileo-combined case do not provide ASRs larger than 99.9% in more than 8 h of the test day, while combining all the four systems allows representative ambiguity-fixed standard deviations smaller than 2 cm in above 70% of the area.

## 5. Real Data Analysis

In this section, making use of the 1 Hz data collected from the two baselines introduced in Section 3 on the test day DOY 224, 2018, the ASRs and the standard deviations of the baseline errors are computed for different system combinations and elevation masks. We remark that the QZSS satellite J07, which was not contained in the MGEX broadcast ephemeris on this day, was not used for the real data analysis. Also note that some Galileo and QZSS satellites were not completely tracked at the beginning of the rising by tens of minutes.

In this study, the empirical IB ASR is given as

(11) $$P_{E}=\frac{l_{C}}{l},$$

where *l* and $l_{C}$ represent the total number of epochs and the number of epochs with ambiguities correctly fixed. In Table 2, the average formal and empirical IB ASRs of baselines CUAA-CUCC and CUAA-CUBB are given with the elevation mask varying from 10 to 40 degrees. The same criteria as for Figure 7a were used to compute representative results. From Table 2, we see that, with an elevation mask of 10 degrees, the combined solutions provide ASRs above 90%. Under an elevation mask of 40 degrees, combining all the four systems still delivers ASRs higher than 95%.

**Figure 11:**
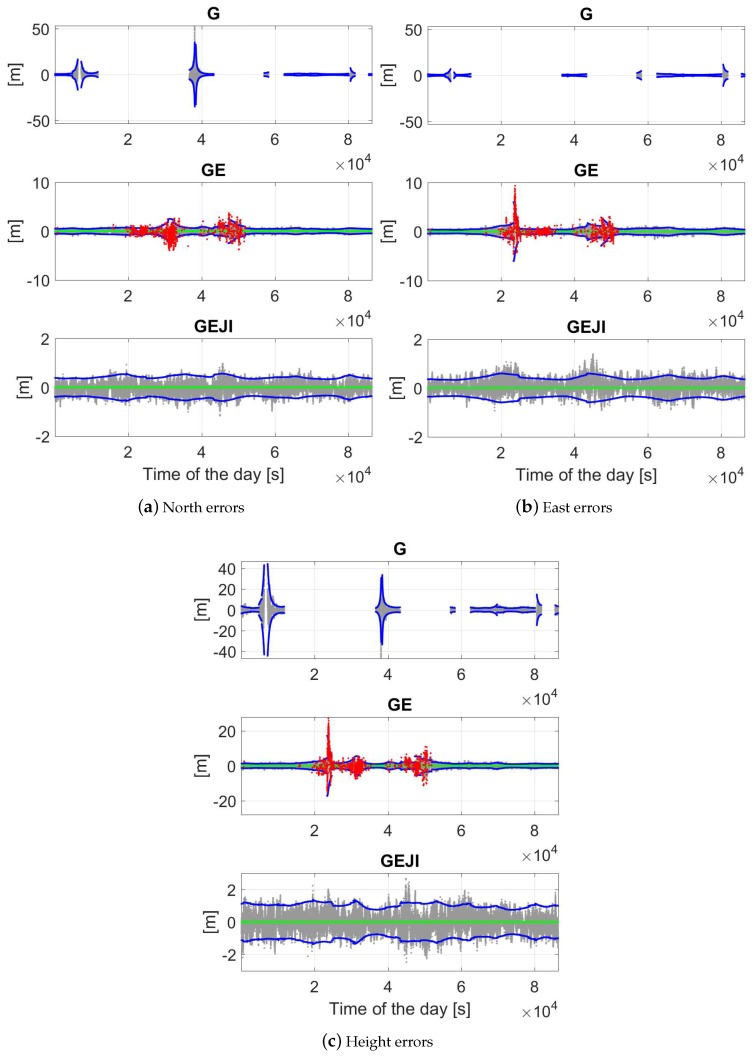
Baseline errors in the north (**a**); east (**b**); and height (**c**) directions with the elevation mask of 10 degrees. Data for baseline CUAA-CUCC on DOY 224, 2018 was used. The gray and blue dots are the ambiguity-float solutions and their 95% formal confidence intervals, respectively. For combined solutions, the green and red dots illustrate the ambiguity-correctly-fixed and -wrongly-fixed solutions, respectively. Note that the scales of the sub-figures are different.

Using the L5 code and phase signals from different system combinations, the baseline errors are illustrated in Figure 11 in the north, east and height directions for baseline CUAA-CUCC on the same day. An elevation mask of 10 degrees was set for the plots, and only the time epochs with at least four satellites available and with PDOP smaller than 100 were used for the plots. The gray, green and red dots represent the ambiguity-float, -correctly-fixed and -wrongly-fixed solutions, respectively. The blue dots illustrate the 95% formal confidence interval of the float solutions. For the GPS-only case (see the top panels), the ambiguity-fixed solutions are not shown due to the low ASR (Table 2). From Figure 11, it can be observed that an increasing number of the utilised systems is helpful to improve both the positioning accuracy and the ASRs. The frequently appeared red dots in GPS/Galileo-combined solutions (the middle panels) around 2–3.5 × 10${}^{4}$ and 4–5.2 × 10${}^{4}$ s correspond to the large ADOPs in these time intervals (see the green dots in Figure 5a). Taking the G/E/J/I-combined solutions as an example (bottom panels), the float solutions (gray dots) are within the 95% formal confidence intervals (blue dots) in 96.1%, 91.3% and 95.8% of the time in the north, east and up directions, respectively. Combining all the four systems, the ambiguity-float baseline errors are within ±1 m in about 95% of the time in all three of the directions.

**Figure 12:**
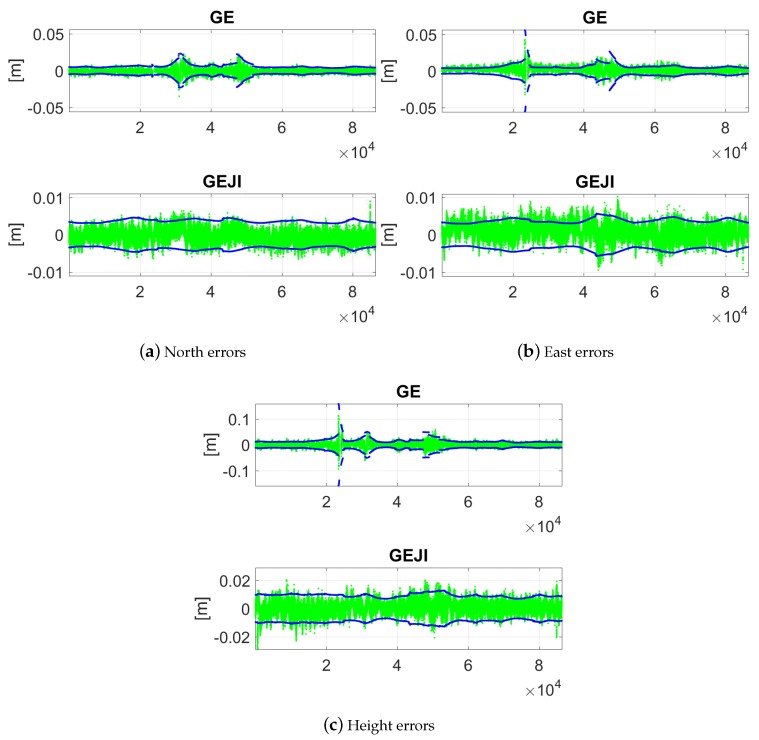
Ambiguity-fixed baseline errors in the north (**a**); east (**b**); and height (**c**) directions with the elevation mask of 10 degrees. Data for baseline CUAA-CUCC on DOY 224, 2018 was used. The green and blue dots are the ambiguity-correctly-fixed solutions and their 95% formal confidence intervals, respectively. Note that the scales of the sub-figures are different.

For the combined cases, the figures are zoomed in to show the ambiguity-correctly-fixed solutions and their 95% formal confidence intervals (Figure 12). Combining all the four systems, in above 95% of the time, the ambiguity-fixed solutions are within ±1 cm in all the three directions.

**Figure 13:**
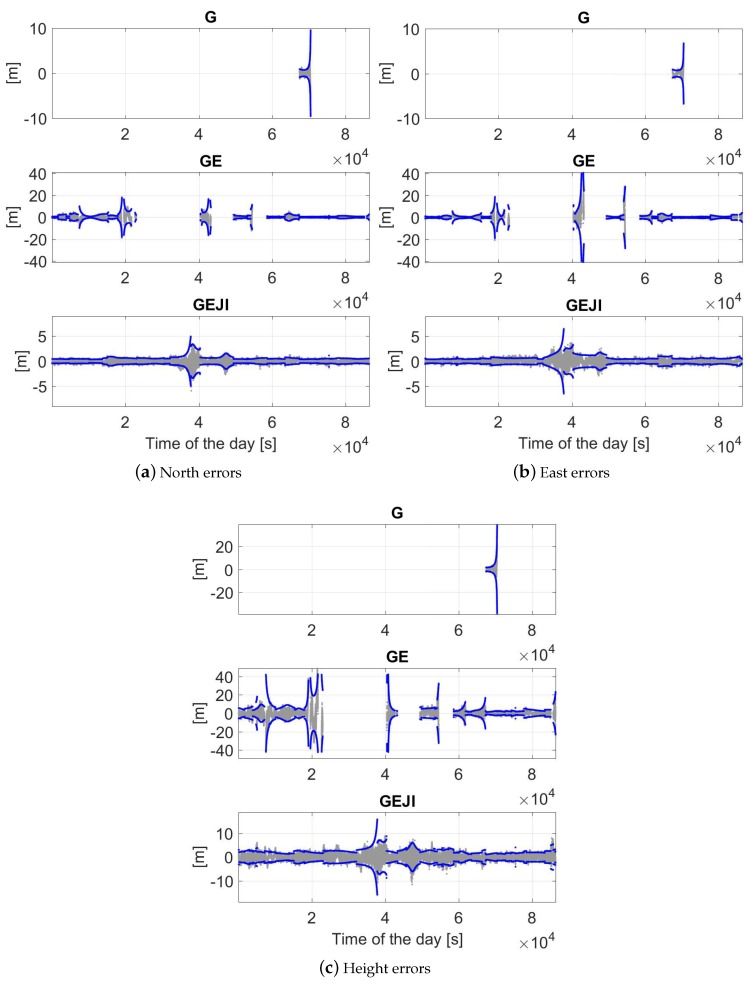
Ambiguity-float baseline errors in the north (**a**); east (**b**); and height (**c**) directions with the elevation mask of 40 degrees. Data for baseline CUAA-CUCC on DOY 224, 2018 was used. The gray and blue dots are the ambiguity-float solutions and their 95% formal confidence intervals, respectively. Note that the scales of the sub-figures are different.

When increasing the elevation mask, worse data availability and positioning accuracy can be observed in all the three directions. Figure 13 shows the baseline errors of CUAA-CUCC on the same day with an elevation mask of 40 degrees. Only the ambiguity-float solutions and their 95% formal confidence intervals are plotted in the figure. We see that, for GPS-only solutions, most of the time within the test day, either less than four satellites can be observed, or the PDOP value is larger than 100. Combining all four systems, in contrast, provides the L5 positioning capability of the entire day. The horizontal (north and east) and the vertical float solutions are within ±1 m in around 95% and 59% of the time, respectively.

**Figure 14:**
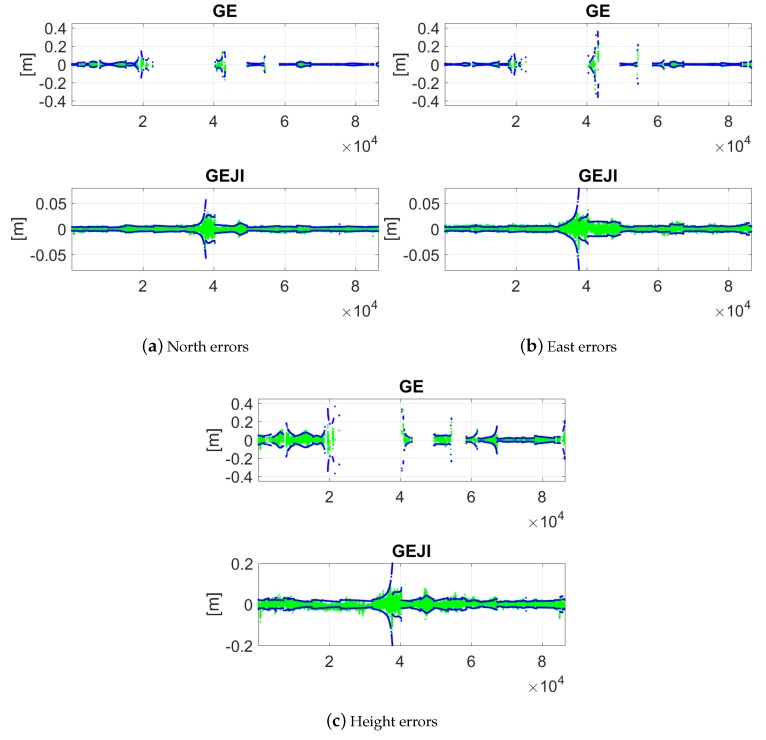
Ambiguity-fixed baseline errors in the north (**a**); east (**b**); and height (**c**) directions with the elevation mask of 40 degrees. Data for baseline CUAA-CUCC on DOY 224, 2018 was used. The green and blue dots are the ambiguity-correctly-fixed solutions and their 95% formal confidence intervals, respectively. Note that the scales of the sub-figures are different.

As comparison to Figure 12, the ambiguity-fixed solutions are also shown in Figure 14 for baseline CUAA-CUCC in the combined cases under the elevation mask of 40 degrees. We see that increasing the elevation mask in general decreases the length of the valid time points and increases the baseline errors. When combining all four of the systems, the baseline errors are within ±1 cm in all three directions about 67% of the time.

The empirical and average formal standard deviations of the ambiguity-float and -fixed solutions are listed in Table 3 and Table 4 for both baselines using different system combinations and elevation masks. Note that the time epochs used for the computation have at least four available satellites and PDOPs smaller than 100. In addition to that, for ambiguity-fixed solutions, only the time epochs with ambiguities correctly fixed are used. As for the ASRs, if the usable data samples are shorter than 8 h, the results are considered to be not representative and are not shown in the tables. From Table 3, it can be observed that, under an elevation mask of 10 degrees, using satellites from combined systems reduces the standard deviations of the ambiguity-float solutions from meter to dm-level in most cases. Under a high elevation mask of 40 degrees, using combined systems is necessary to obtain positioning results longer than 8 h within the test day. The standard deviations in such cases are at a dm-to-meter level.

In the ambiguity-fixed case, only the valid time points with correctly-fixed ambiguities contribute to the standard deviations, and the 8 h lower limit is defined as before to deliver representative results. Note that the percentage of the time points with correctly fixed ambiguities should correspond to the empirical ASR or the average formal ASR in Table 2, but not the percentage of the time points with the formal ASR larger than 99.9%. This explains, e.g., that, in GPS/Galileo-combined case with an elevation mask of 40 degrees, no representative ambiguity-fixed standard deviations were shown in the middle bottom panel of Figure 10, but the empirical ASR or the percentage of the time points with correctly fixed ambiguities is still above 30% (8 h), so that the ambiguity-fixed standard deviations are listed in Table 4 in such case. From Table 4, we see that combining all the four systems delivers ambiguity-fixed standard deviations at mm-level under an elevation mask of 10 degrees based on solutions of the entire day. Under an elevation mask of 40 degrees, ambiguity-fixed standard deviations at mm-to-cm level can be obtained based on time points in more than 95% of the test day, when all the four systems are combined.

## 6. Receivers with Larger Signal Standard Deviations

The L5 multi-GNSS RTK positioning is not only limited to geodetic receivers. To explore the potential of the L5 RTK positioning of low-cost receivers using all the available satellites sending signals on 1176.45 MHz, and also to simulate the situation in environments with larger multipaths, we compute the average formal standard deviations of the baseline errors with the varying code and phase signal standard deviations based on the location of Perth. In this study, analysis is performed for code standard deviations varying from 3 dm to 1 m and phase standard deviations varying from 3 mm to 1 cm. The signal standard deviations are assumed to be equal for all systems. Note that, for the simulations on the test day DOY 224, 2018, as in Section 4, the positions (WGS84) of J07 were taken from DOY 154, 2018 for the completeness of the QZSS constellations. To differ from the computation based on the signal standard deviations in Table 1, station coordinates of CUAA and CUCC are used with the names changed to CUAU and CUCU.

**Figure 15:**
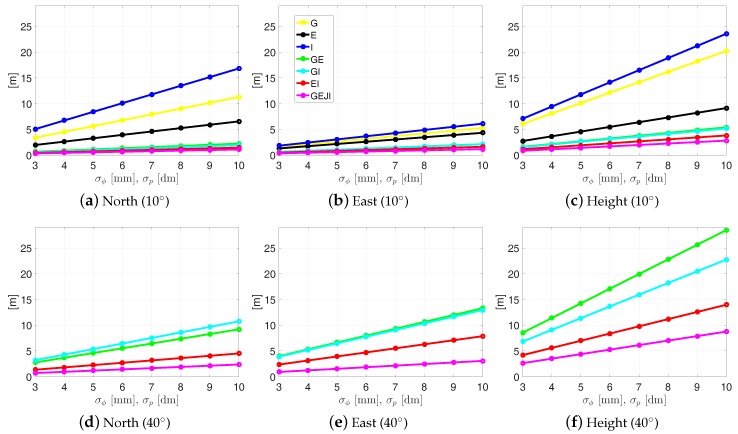
Average ambiguity-float formal standard deviations of the baseline errors for CUAU-CUCU on DOY 224, 2018. The elevation mask was set to be 10 (**a**–**c**) and 40 degrees (**d**–**f**). Note that the sub-figures have different scales.

Figure 15 shows the average ambiguity-float formal standard deviations of CUAU-CUCU with varying L5 code and phase standard deviations. Note that, here, all the time epochs with at least four satellites observable and with PDOP smaller than 100 were used to compute the average formal standard deviations in the ambiguity-float case. The 8 h lower time limit is set as before to compute representative results. From Figure 15, we see that combining different systems is important for reducing the formal standard deviations for both low and high elevation masks. Under an elevation mask of 40 degrees, representative results can only be computed in a combined case, and combining all the four systems (see the magenta lines in the bottom panels) deliver meter-level average formal standard deviations even with large phase and code standard deviations of 1 cm and 1 m, respectively.

In the ambiguity-fixed case, only the valid time points (with at least 4 satellites and PDOP smaller than 100) with ASR larger than 99.9% were used, and a 8 h lower time limit is set as before. As shown in Table 5, in our tested system combinations, only E/I and G/E/J/I-combined cases deliver ambiguity-fixed results for more than 8 h within the test day, provided the signal standard deviations are small enough. Under a high elevation mask of 40 degrees, this applies only to the G/E/J/I-combined case with the smallest signal standard deviations in our tests, i.e., 3 mm for phase and 3 cm for code. The average formal ambiguity-fixed standard deviations are at mm-level in the horizontal directions, and at mm-to-cm level in the vertical direction depending on the signal standard deviations.

## 7. Conclusions

Nowadays, the precise L5 signal on frequency 1176.45 MHz is sent by an increasing number of satellites from different GNSSs. In this study, investigations were performed for L5-only single-epoch multi-GNSS RTK positioning under different elevation masks. It is verified that combining satellites from current constellations of GPS IIF, Galileo, IRNSS and QZSS allows L5 instantaneous RTK positioning even in constrained measurement environments, where a high elevation mask is required.

Based on the signal standard deviations obtained from two baselines in Perth, Australia, formal analysis is performed for baselines in Perth and a larger area covering Australia, part of the Indian Ocean, the Pacific Ocean and Asia. Using the real data collected from two baselines in Perth, empirical analysis is also performed considering the fact that less signals are tracked than in the simulations. For the baselines in Perth, while the single-system mean ASR is lower than 50% even under a nominal elevation mask of 10 degrees, combing all the four systems (GPS/Galileo/QZSS/IRNSS) delivers ASR above 95% under a high elevation mask of 40 degrees. Under the elevation mask of 40 degrees, in GPS/Galileo/QZSS/IRNSS-combined case, high average ASR above 95% can also be achieved for other baselines located in most of Australia and southeast Asia. For the baselines in Perth, using single-system L5 signals, the poor geometry under a high elevation mask of 40 degrees hampers meaningful positioning results of more than 8 h within a day even without ambiguity resolution. However, combining all the four systems allows ambiguity-fixed and -float solutions with a standard deviation at mm-to-cm and dm-to-meter level, respectively, based on solutions of almost the entire test day even with the elevation cut-off angle of 40 degrees. Average formal standard deviations at these levels can also be achieved in most of Australia and southeast Asia.

Analysis was also performed for receivers in Perth with larger signal standard deviations, i.e., for low-cost receivers or receivers located in environments with larger multipaths. Tests with varying signal standard deviations show that meaningful ambiguity-float solutions of more than 8 h within the test day can only be delivered in combined case, if a high elevation mask of 40 degrees is set. In ambiguity-fixed case, in our tested system combinations, only combining all the four systems delivers ambiguity-fixed results of more than 8 h within the day under the elevation mask of 40 degrees, provided the signal standard deviations are small enough, i.e., 3 mm and 3 dm for phase and code, respectively.

## Figures and Tables

**Table 1:** L5 multipath-uncorrected phase ($\sigma_{\phi }$) and code standard deviations ($\sigma_{p}$) for GPS, Galileo, QZSS and IRNSS satellites. The standard deviations are given for elevation masks of 10/40 degrees. The data on DOY 223 was used for computing the standard deviations for GPS, Galileo and IRNSS, and the data on DOY 153, 2018 was used for computing the QZSS standard deviations.

System	CUAA-CUCC		CUAA-CUBB	
	$\sigma_{\phi }$ [mm]	$\sigma_{p}$ [cm]	$\sigma_{\phi }$ [mm]	$\sigma_{p}$ [cm]
GPS	2/1	17/15	2/1	17/14
Galileo	2/1	19/17	2/1	18/15
QZSS	2/2	14/13	2/2	16/15
IRNSS	2/1	25/21	2/1	26/26

**Table 2:** Empirical and average formal IB ASRs (in brackets). The data of baseline CUAA-CUCC and CUAA-CUBB on DOY 224, 2018 was used.

System ID	CUAA-CUCC			CUAA-CUBB		
**Elevation Mask**	**10** ${}^{\circ }$	**25** ${}^{\circ }$	**40** ${}^{\circ }$	**10** ${}^{\circ }$	**25** ${}^{\circ }$	**40** ${}^{\circ }$
G	0.151(0.130)	–	–	0.160(0.132)	–	–
E	0.458(0.411)	0.129(0.128)	–	0.484(0.441)	0.158(0.151)	–
I	0.050(0.066)	0.041(0.064)	–	0.058(0.059)	0.054(0.055)	–
G/E	0.925(0.906)	0.692(0.664)	0.489(0.457)	0.915(0.909)	0.687(0.682)	0.516(0.488)
G/I	0.925(0.946)	0.752(0.766)	0.193(0.200)	0.928(0.942)	0.759(0.762)	0.168(0.186)
E/I	0.997(1.000)	0.962(0.972)	0.494(0.487)	1.000(1.000)	0.959(0.975)	0.491(0.485)
G/E/J/I	1.000(1.000)	0.999(0.997)	0.962(0.958)	1.000(1.000)	1.000(1.000)	0.969(0.951)

**Table 3:** Empirical and average formal (in brackets) standard deviations of the ambiguity-float baseline errors. The results are given in the north/east/height directions. The data on DOY 224, 2018 was used for both baselines.

System ID	CUAA-CUCC [dm]		CUAA-CUBB [dm]	
**Elevation Mask**	**10** ${}^{\circ }$	**40** ${}^{\circ }$	**10** ${}^{\circ }$	**40** ${}^{\circ }$
G	22(19)/7(9)/31(34)	–	18(19)/7(9)/30(35)	–
E	9(13)/9(9)/21(21)	–	11(12)/9(8)/20(20)	–
I	37(41)/14(15)/51(58)	–	43(44)/16(16)/60(61)	–
G/E	4(4)/5(5)/13(12)	16(15)/21(21)/48(45)	4(4)/4(5)/11(12)	14(14)/24(20)/38(43)
G/I	4(4)/5(5)/11(11)	24(20)/21(23)/47(45)	4(4)/5(5)/13(12)	29(24)/26(27)/55(53)
E/I	3(3)/4(3)/7(8)	7(9)/11(15)/27(28)	3(3)/4(3)/8(8)	8(10)/13(17)/31(32)
G/E/J/I	2(2)/2(2)/5(5)	4(4)/5(5)/14(15)	2(2)/2(2)/5(5)	4(5)/5(6)/15(17)

**Table 4:** Empirical and average formal (in brackets) standard deviations of the ambiguity-fixed baseline errors. The results are given in the north/east/height directions. The data on DOY 224, 2018 was used for both baselines.

System ID	CUAA-CUCC [mm]		CUAA-CUBB [mm]	
**Elevation Mask**	**10** ${}^{\circ }$	**40** ${}^{\circ }$	**10** ${}^{\circ }$	**40** ${}^{\circ }$
E	3(4)/4(4)/9(10)	–	3(4)/4(4)/9(10)	–
G/E	3(3)/3(4)/7(9)	6(8)/8(9)/19(24)	3(3)/3(3)/8(8)	6(7)/8(8)/18(22)
G/I	3(3)/3(3)/7(8)	–	3(3)/3(3)/7(8)	–
E/I	2(2)/3(3)/6(6)	3(4)/5(6)/16(19)	2(2)/3(3)/6(6)	3(4)/4(5)/15(16)
G/E/J/I	2(2)/2(2)/5(5)	3(4)/3(5)/11(13)	2(2)/2(2)/4(5)	3(3)/3(4)/10(12)

**Table 5:** Average ambiguity-fixed formal standard deviations of the baseline errors for CUAU-CUCU in Galileo/IRNSS and GPS/Galileo/QZSS/IRNSS-combined cases. The MGEX combined broadcast ephemeris on DOY 224, 2018 was used for the computation. The standard deviations (STD) are given in the format of North/East/Up directions.

Signal STD	E/I [mm]	G/E/J/I [mm]
**Elevation Mask**	**10** ${}^{\circ }$	
$\sigma_{\phi }$ = 3 mm, $\sigma_{p}$ = 3 dm	4/5/12	3/3/8
$\sigma_{\phi }$ = 4 mm, $\sigma_{p}$ = 4 dm	–	4/5/11
$\sigma_{\phi }$ = 5 mm, $\sigma_{p}$ = 5 dm	–	5/6/14
Elevation mask	40${}^{\circ }$	
$\sigma_{\phi }$ = 3 mm, $\sigma_{p}$ = 3 dm	–	4/5/19

## Appendix A

Table A1 and Table A2 list the main variables and acronyms in this paper.

**Table A1:** Table of variables.

Name	Meaning
Δϕ	Phase O-C term
Δp	Code O-C term
$D_{m}$	Differencing operator
$u^{s}$	Satellite (*s*)-to-receiver unit vector
$\lambda_{j}$	Wavelength on frequency *j*
Δb	Baseline increment vector
*a*	Ambiguity vector
$Q_{\phi }$	Variance-covariance matrix of the phase observations
$Q_{p}$	Variance-covariance matrix of the code observations
$w^{s}$	Weighting function for satellite *s*
${\mathrm{ele}}^{s}$	Elevation angle from receiver to satellite *s*
$\sigma_{\phi }$	Phase signal standard deviation in the zenith direction
$\sigma_{p}$	Code signal standard deviation in the zenith direction
$\sigma_{{\hat{z}}_{i|K}}$	The *i*-th conditional standard deviation of the decorrelated ambiguity with K=1,⋯,i−1
$P_{ILS}$	Formal ILS ASR
$P_{IB}$	Formal IB ASR
$P_{E}$	Empirical IB ASR
$Q_{\hat{a}\hat{a}}$	Variance-covariance matrix of the float ambiguities

**Table A2:** Table of acronyms.

Acronyms	Full Name
RTK	Real time kinematic
ASR	Ambiguity success rate
IRNSS	Indian regional navigation satellite system
IGSO	Inclined geosynchronous orbit
GEO	Geostationary orbit
QZSS	Quasi-zenith satellite system
QZO	Quasi-zenith orbit
IOV	In-orbit validation
FOC	Full operational capability
DOY	Day of year
MGEX	Multi-GNSS experiment
DD	Double-difference
O-C	Observed-minus-computed
ISB	Inter-system bias
PDOP	Position dilution of precision
IB	Integer bootstrapping
ILS	Integer least-squares
ADOP	Ambiguity dilution of precision
LAMBDA	Least-squares ambiguity decorrelation adjustment
